# High-Density Functional Near-Infrared Spectroscopy and Machine Learning for Visual Perception Quantification

**DOI:** 10.3390/s23218696

**Published:** 2023-10-25

**Authors:** Hongwei Xiao, Zhao Li, Yuting Zhou, Zhenhai Gao

**Affiliations:** 1 School of Automotive Engineering, Jilin University, Changchun 130022, China; xiaohw@jlu.edu.cn; 2 School of Public Health, Jilin University, Changchun 130021, China; liz@jlu.edu.cn; 3 China Academy of Engineering Physics, Mianyang 621900, China; zhouyuting21@gscaep.ac.cn; 4 State Key Laboratory of Automotive Simulation and Control, Jilin University, Changchun 130025, China

**Keywords:** visual perception, functional near-infrared spectral imaging techniques, statistics, machine learning, information theory

## Abstract

The main application scenario for wearable sensors involves the generation of data and monitoring metrics. fNIRS (functional near-infrared spectroscopy) allows the nonintrusive monitoring of human visual perception. The quantification of visual perception by fNIRS facilitates applications in engineering-related fields. This study designed a set of experimental procedures to effectively induce visible alterations and to quantify visual perception in conjunction with the acquisition of Hbt (total hemoglobin), Hb (hemoglobin), and HbO_2_ (oxygenated hemoglobin) data obtained from HfNIRS (high-density functional near-infrared spectroscopy). Volunteers completed the visual task separately in response to different visible changes in the simulated scene. HfNIRS recorded the changes in Hbt, Hb, and HbO_2_ during the study, the time point of the visual difference, and the time point of the task change. This study consisted of one simulated scene, two visual variations, and four visual tasks. The simulation scene featured a car driving location. The visible change suggested that the brightness and saturation of the car operator interface would change. The visual task represented the completion of the layout, color, design, and information questions answered in response to the visible change. This study collected data from 29 volunteers. The volunteers completed the visual task separately in response to different visual changes in the same simulated scene. HfNIRS recorded the changes in Hbt, Hb, and HbO_2_ during the study, the time point of the visible difference, and the time point of the task change. The data analysis methods in this study comprised a combination of channel dimensionality reduction, feature extraction, task classification, and score correlation. Channel downscaling: This study used the data of 15 channels in HfNIRS to calculate the mutual information between different channels to set a threshold, and to retain the data of the channels that were higher than those of the mutual information. Feature extraction: The statistics derived from the visual task, including time, mean, median, variance, extreme variance, kurtosis, bias, information entropy, and approximate entropy were computed. Task classification: This study used the KNN (K-Nearest Neighbors) algorithm to classify different visual tasks and to calculate the accuracy, precision, recall, and F1 scores. Scoring correlation: This study matched the visual task scores with the fluctuations of Hbt, Hb, and HbO_2_ and observed the changes in Hbt, Hb, and HbO_2_ under different scoring levels. Mutual information was used to downscale the channels, and seven channels were retained for analysis under each visual task. The average accuracy was 96.3% ± 1.99%; the samples that correctly classified the visual task accounted for 96.3% of the total; and the classification accuracy was high. By analyzing the correlation between the scores on different visual tasks and the fluctuations of Hbt, Hb, and HbO_2_, it was found that the higher the score, the more obvious, significant, and higher the fluctuations of Hbt, Hb, and HbO_2_. Experiments found that changes in visual perception triggered changes in Hbt, Hb, and HbO_2_. HfNIRS combined with Hbt, Hb, and HbO_2_ recorded by machine learning algorithms can effectively quantify visual perception. However, the related research in this paper still needs to be further refined, and the mathematical relationship between HfNIRS and visual perception needs to be further explored to realize the quantitative study of subjective and objective visual perception supported by the mathematical relationship.

## 1. Introduction

fNIRS is a technology that indirectly reflects the level of brain activity by detecting changes in the concentration of hemoglobin in the brain; this technology has facilitated the in-depth exploration of the cognitive functions of brain regions. The brain needs to perform oxygenation activities in different perceptual states, and changes in the concentration of HbO_2_ and Hb can characterize the intensity of brain activity [1]. fNIRS can quantify the issues that trigger brain activity and help researchers explore the mysteries of the human brain.

fNIRS is easy to operate and has high temporal resolution, theoretically up to milliseconds. It does not require slicing and has no substantial fluorescence interference; it can be adapted to in situ localization of fresh tissues; it is resistant to motion interference and electromagnetic interference and has a reasonable prospect of application in different fields of research [2]. However, conventional fNIRS has limitations, including significantly lower spatial resolution than functional MRI (magnetic resonance imaging), and the depth of measurement is concentrated in the superficial cortex of the brain. HfNIRS employs high-density diffuse optical tomography to provide channels of different lengths spanning short-range and long-range, thus providing higher spatial resolution, more depth information, and better signal quality.

Visual perception is generated by “seeing”, which is both objective and subjective [3]. From a physiological point of view, visual perception refers to the process in which light strikes the human eye and the optical system is stimulated to form a visible image [4]. From a psychological point of view, visual perception can also be understood by thinking [5]: the viewer analyzes and thinks about the information collected by the optical system and finally forms an understanding. Visual perception also influences the organization and knowledge of external knowledge through other perceptions [6], assisting people in perceiving stimuli from various elements of the environment and in building an understanding of the external environment. The quantification of visual perception helps to guide technological innovation and application generalization across industries [7]. In the process of dynamic change, the participants’ visual perception represents the visual feedback for the current scenario [8]. Traditional research methods focus on quantifying visual perception through questionnaires after the participants experience dynamic visual changes [9]. Due to the subjectivity of the questionnaire responses [10], the results obtained by the questionnaire alone have low credibility, but the questionnaire is the critical means of quantifying the problem.

To solve the above problems, HfNIRS was used in this study to collect the fluctuations of Hbt, Hb, and HbO_2_ in the participants during visual changes. In this study, a car driving scenario was simulated in which the brightness and saturation of the car’s operating interface changed during the driving process. In response to the visual changes, the participants completed the layout, color, design issues, and information questions. In the data analysis step, Step 1 calculates the mutual information between channels, retaining channels higher than the threshold and achieving channel dimensionality reduction to avoid dimensional disaster in high-dimensional data analysis. Step 2 employs feature engineering to extract multiple statistical features of different problems. Step 3 uses characteristics and question tags in a data input classification algorithm and calculates the classification results of the evaluation index. Step 4 explores the corresponding relationship between the fluctuations of Hbt, Hb, and HbO_2_ and the scores of different questions in the questionnaire to realize the quantification of visual perception in the interaction behavior of the human–computer interface and to avoid the low credibility of the experimental results caused by the misjudgment of the subjective questionnaire.

There are three main innovations in this work:

1. Using HfNIRS and the quantitative questionnaire to combine the visual guide, man–machine interactive interface design, information, and other problems in creating the layout, color, and visual perception.
2. Using high spatial-resolution data from HfNIRS to quantify vision.
3. Implementing the whole process analysis of HfNIRS based on statistics, information theory, and machine learning algorithms.

This paper comprises the following five parts: Section 2 introduces the related theories; Section 3 describes the experimental equipment, experimental process, signal processing, feature engineering, and classification algorithm; Section 4 describes the results; Section 5 contains the discussion; Section 6 provides the conclusion and prospects.

## 2. Related Work

Visual perception is classified as static or dynamic based on the motion relationship between scenes. Static perception means that the location is presented as stationary and perpendicular to the viewer’s line of sight, and the observer and the setting are relatively inactive. The static visual perception process is two-dimensional, and most scenes and viewpoints are fixed; so, it is difficult for observers to observe the scene in a comprehensive way. Dynamic perception means that the viewer is free to follow objects without the constraints of the stage. The observer feels the dynamic changes of the scene in the continuous movement and perceives the active and ongoing visual impression. The dynamic visual perception process is three-dimensional. With the transformation of the stage, the line of sight and angle of view also change, achieving the effect of “moving the scene” and forming a holistic cognition of the external environment. Dynamic and static perceptions can be carried out independently, but they often cooperate to form the overall perception. Static perception needs a series of emotional perceptions to enrich the perception process. In contrast, dynamic perception needs static perception as an essential node to highlight the key points and to improve the perception process during rhythm changes.

There are various ways to analyze visual perception, and the current visual perception analysis methods mainly include questionnaires, model construction, and physiological electrical signal analysis. It is one of the standard methods to analyze visual perception from the perspective of the questionnaire. Bastard used the QUEPAHVA questionnaire to quantify the perception of visual disorders in adults and further verified psychological problems [11]. Yuan used a combination of questionnaire survey, interview, and logistic regression to quantify visual perception from the perspective of urban development [12]. Jae Hoon Ma designed nine cases of IVE(International Videotex Equipment) lighting and analyzed participants’ responses to visual perception and task performance through questionnaires and Landolt C tests [13]. Given the problems in questionnaire analysis, model construction has been widely developed in recent years. Jia used a three-dimensional quantitative model to quantify the visual perception of highway space and put forward the promotion strategy of landscape construction [14]. Xiang used the satisfaction model of visual and auditory perception combined with principal component analysis, correlation analysis, and linear regression to realize the visual perception analysis of urban green space [15]. Li collected autonomous driving data from the visual perceptions of drivers and proposed an IROL interpretable prediction model for curved segments of two-lane rural roads [16]. With the continuous development of portable devices for collecting physiological electrical signals, the quantification of visual perception is gradually being carried out from the perspective of physiological electrical signals. Li took Xiamen University as the research object, summarized the visual preferences of tourists using network big data, selected eye movement experimental stimuli for eye movement exploratory analysis, and analyzed the visual perceptions of tourists in combination with questionnaire data [17]. Fan examined the changes in visual perception in VR (Virtual Reality) scenes by collecting EEG (electroencephalogram) data [18]. Jose analyzed the changes in visual perception in patients with liver cirrhosis in the early stage by recording the electroencephalograms of 89 patients with liver cirrhosis [19].

fNIRS is an emerging non-invasive brain neuroimaging technique with broad application prospects; it has been increasingly used in neuroscience research in recent decades. The principle of fNIRS is as follows: The blood oxygen content of local tissues in the human body changes to a certain extent with the metabolic activities of human organs and tissues. The specificity of hemoglobin’s absorption of near-infrared light leads to changes in the light flux reflected by near-infrared light. Therefore, the incident light intensity is known using near-infrared light to illuminate human tissues and the use of a receiver to detect the light intensity. According to the Beer–Lambert law, the hemodynamic activity of the local brain tissue in the cerebral cortex can be seen in real time, efficiently and directly [20]. In fNIRS experiments, researchers use fNIRS to observe changes in the concentration of HbO_2_ and Hb in selected brain regions if the hemodynamic activity in those regions is highly relevant to the task design. It can be inferred that the experimental task activates this brain area [21].

fNIRS has been applied in research in many fields. Zhou collected the fNIRS of 28 participants, and the repeated measurement method was used to analyze the audio perception of the participants [22]. Zhou collected fNIRS data from 75 participants and used correlation analysis to explore the emotional perception of the participants [23]. Chen collected fNIRS and EEG data from 20 volunteers and analyzed the tactile perception of the participants by the Mel frequency cepstral coefficient [24]. Mazziotti proposed that the amplitude of fNIRS responses in visual cortex revealed typical autistic features in children, and the paper showed that animal-based stimuli could evoke visual responses in adult cerebral cortex. It is possible to quantify visual perception by fNIRS [25].

The existing research has yet to quantify visual perception from the perspective of the combination of HfNIRS and questionnaire data. This study uses HfNIRS and questionnaire data to quantify visual perception and put forward quantitative indicators.

## 3. Materials and Methods

### 3.1. Data Collection

**Figure 1:**
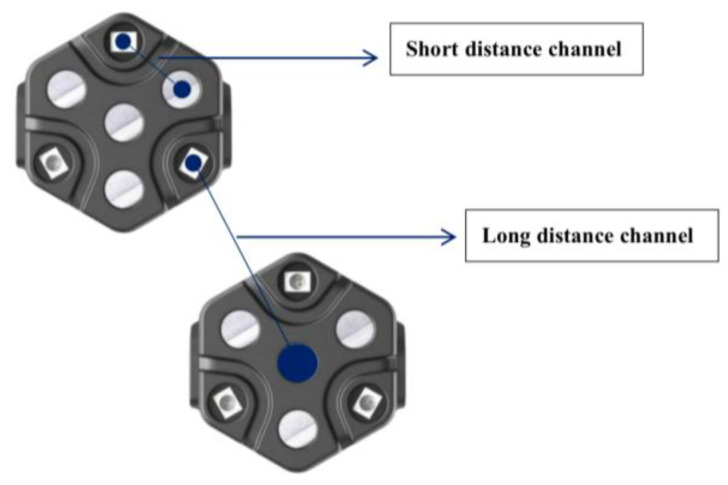
HfNIRs short-range and long-range channels.

**Figure 2:**
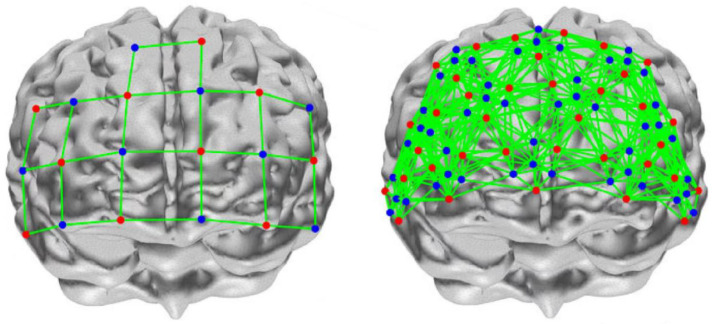
Conventional fNIRs density sampling is shown on the left, and HfNIRs high-density sampling is shown on the right. Each colored dot represents the device sensor site.

Some things could be improved in the related research on visual perception. To ensure the accuracy of the experimental study, the UCL (University College London) wearable HfNIRS was used for data acquisition. The system configuration contains 16 sites; each site contains three light source sensors and four detector sensors. Compared with conventional fNIRS, data from up to 48 to 1728 channels can be obtained simultaneously, and the same site is measured as a short-channel signal. Short-channel signals allow the measurement of scalp signals. Long-channel signals can be measured in adjacent areas. Long-channel signals enable the measurement of brain and scalp signals. Reliable brain measurements can only be obtained by the regression analysis of short-channel signals and long-channel signals (Figure 1). High-density sampling increases the range of brain regions measured, facilitating the acquisition of more reliable brain function data, and flexible configuration reduces the time needed for the experiment (Figure 2).

**Figure 3:**
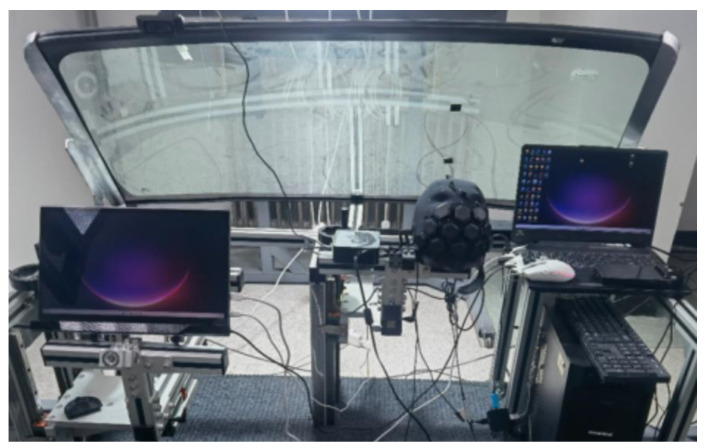
The driving environment was simulated, with the participant driving scene on the left and the experimenter experimental scene on the right.

The cockpit was constructed using a self-built simulation bench for simulating the driving environment. The main configuration of the self-built simulation bench includes the main frame, cab floor, seat adjustment and installation interface, instrument display platform, central control display platform, direction control platform, and glass support platform. This platform is a professional man–machine platform language for light and color research. The adjustment range of each functional part covers the whole range of passenger cars. The height of the H point is 100–450 mm, and the adjustment range of the seat front and rear is 500–900 mm. The center height of the steering wheel is adjusted to 600~750 mm, the front and back adjustment range is 200~450 mm, and the angle adjustment range is 10~30 degrees. The experimental screen was a 15.6-inch display device with a 100% color gamut, and the position was adjusted to the corresponding part of the actual car display screen to ensure the accuracy of the experiment. The standard D65 light source was used in the experiment (Figure 3).

### 3.2. Basic Information of Participants

The questionnaire and functional near-infrared spectroscopy data were collected from 29 volunteers, including 19 males and 10 females, all of whom had more than one year of driving experience, were fluent in the language, and had no mental disorders or regular use of drugs. Informed consent was obtained from all the volunteers before participation in the experiment, and the experimental procedures followed the ethical requirements of the Ethics Committee of Jilin University.

### 3.3. Data Acquisition Procedures

The experiment included two parts: the pre-experiment and the formal experiment, to ensure the validity and accuracy of the experimental results. In the pre-experiment, the participants were required to complete one investigation to obtain proficiency in the practical operation. If they needed to become more familiar with the testing procedure, they could choose to practice again. The practice phase questions were the same as those of the experimental phase. However, the practice phase pictures differed from the testing phase pictures to prevent the repetition of the practice and practical stages.

**Figure 4:**
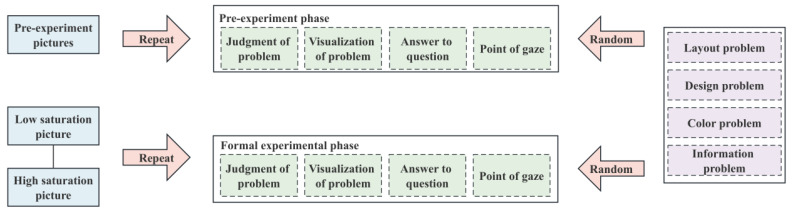
Data acquisition process.

Figure 4 shows the data collection procedure. The experimental phase process was divided into four steps. The first step was the problem judgment stage: a subjective questionnaire appeared on the screen, and the participants needed to focus on the problem. The second part was the picture visualization stage: a random picture emerged after 4000 ms of problem judgment. The third part was the question-answering step: after the image was presented for 6000 ms, an evaluation interface with a white and black background appeared. The fourth stage was the gaze point stage: the gaze calibration rest interface with white and black backgrounds appeared, and the subsequent trial was entered into after 8000 ms.

**Figure 5:**
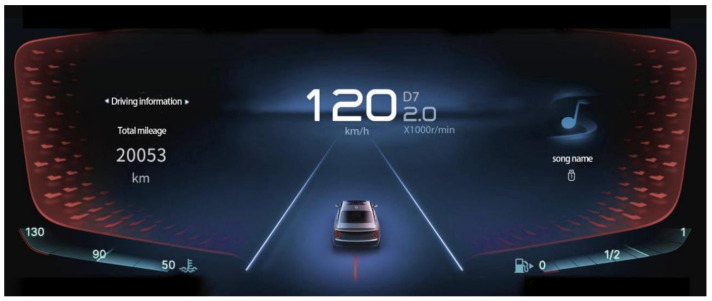
Experimental picture—low saturation.

**Figure 6:**
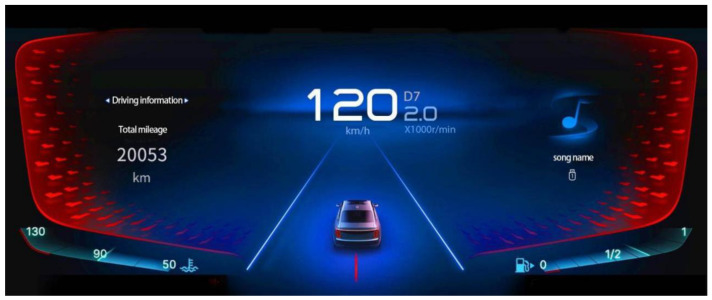
Experimental picture—high saturation.

The experiment consisted of four questions and two kinds of saturation pictures and was repeated randomly. The formal investigation had 2 × 4 × 2 = 16 trials, and the experiment took about 5 min to complete. However, due to the individual differences in each person’s reaction to the pictures, the investigation took about 5–7 min. The four problems were layout, design, color, and information problems. The basic structure of the two saturation pictures was the same; one was a low-saturation picture, as shown in Figure 5, and the other was a high-saturation picture, as shown in Figure 6.

### 3.4. Signal Processing

The acquisition of fNIRS signals requires three light sources and four detector sensors. The emitted radiation travels through the biological tissues of the head, namely the skin, superficial blood vessels, bone, brain, and blood vessels of the brain, reaching the detector by scattering effects. Three different luminescent light sources were used, and their relative concentrations could be inferred from differences in the interaction of the luminescent light source with the chromophore of hemoglobin.

The first step in signal processing is the application of the modified Beer–Lambert law, which converts the detection values into varying chromophores at each concentration, namely HbO_2_ and Hb, given specific parameters, the emission detector distance, and the extinction coefficient at each wavelength. Equations (1) and (2) show the modified Beer–Lambert law used to calculate the relative concentration of each chromophore. DPF is the differential path length factor, $\epsilon_{Hb/HbO_{2}}^{\lambda_{x}}$ is the extinction coefficient of each wavelength close to each chromosphere of HbO_2_, and I represents the light intensity (b at the baseline and t at the task).

(1) $${∆HbO}_{2}=\frac{log\frac{I_{b}^{\lambda_{1}}}{I_{t}^{\lambda_{1}}}\epsilon_{Hb}^{\lambda_{2}}-\frac{I_{b}^{\lambda_{2}}}{I_{t}^{\lambda_{2}}}\epsilon_{Hb}^{\lambda_{1}}}{d\cdot DPF[\epsilon_{HbO_{2}}^{\lambda_{1}}\epsilon_{Hb}^{\lambda_{2}}-\epsilon_{HbO_{2}}^{\lambda_{2}}\epsilon_{Hb}^{\lambda_{1}}]}$$

(2) $$∆Hb=\frac{log\frac{I_{b}^{\lambda_{1}}}{I_{t}^{\lambda_{1}}}\epsilon_{HbO_{2}}^{\lambda_{2}}-\frac{I_{b}^{\lambda_{2}}}{I_{t}^{\lambda_{2}}}\epsilon_{HbO_{2}}^{\lambda_{1}}}{d\cdot DPF[\epsilon_{HbO_{2}}^{\lambda_{1}}\epsilon_{Hb}^{\lambda_{2}}-\epsilon_{HbO_{2}}^{\lambda_{2}}\epsilon_{Hb}^{\lambda_{1}}]}$$

Here, ${∆HbO}_{2}$ represents the relative concentration of HbO_2_, ∆Hb represents the relative concentration of Hb,$\epsilon_{Hb}^{\lambda_{1}}$ represents the relative concentration of each chromophore of Hb at wavelength $\lambda_{1}$,$\epsilon_{HbO_{2}}^{\lambda_{1}}$ represents the relative concentration of each chromophore of HbO_2_ at wavelength $\lambda_{1}$,$\epsilon_{Hb}^{\lambda_{2}}$ represents the relative concentration of each chromophore of Hb at wavelength $\lambda_{2}$,$\epsilon_{HbO_{2}}^{\lambda_{2}}$ represents the relative concentration of each chromophore of HbO_2_ at wavelength $\lambda_{2}$,$I_{b}^{\lambda_{1}}\;$ represents the light intensity at the baseline at wavelength $\lambda_{1}$, $I_{b}^{\lambda_{2}}\;$ represents the light intensity at the baseline at wavelength $\lambda_{2}$, $I_{t}^{\lambda_{1}}$ represents the light intensity during the task at wavelength $\lambda_{1}$, and $I_{t}^{\lambda_{2}}$ represents the light intensity during the task at wavelength $\lambda_{2}$.

The DPF was the same in both cases, so the change in concentration was calculated as a factor. The wavelengths of the embers used in this study were 760 nm (λ_1_) and 850 nm (λ_2_), and the extinction coefficients were calculated according to Matcher. This study calculated the change in Hbt for each chromophore, which is the sum of Hb and HbO_2_, as mentioned earlier. After this conversion, the signal was filtered using a second-order bandpass Butterworth finite filter with a pulse filter cutoff frequency of 0.01 Hz to remove electrical and physiological noise and maintain the most informative frequency band of the HfNIRS signal. According to the experimental process, each dataset was divided into 16 windows, and each window had a label. If it belonged to the period of the layout problem, it was marked as a “layout problem”. This processing yielded windows 32 times for each participant (16 times per baseline period).

### 3.5. Data Preprocessing and Channel Screening

To ensure the validity of the calculation in the data analysis process, the HbO_2_ and Hb data of the 5 channels and 15 sensors calculated in this study were normalized by linear data. Equation (3) shows the normalization method of the linear data. After normalization, the speed of the gradient descent to find the optimal solution can be accelerated, and the accuracy can be improved [26].

(3) $$x^{\prime }=\frac{x-min(x)}{\max\left(x\right)-min(x)}$$

Here, $x^{\prime }$ represents the normalized data vector, x represents the original data vector, min(x) represents the minimum value in the original data vector, and $\max\left(x\right)$ represents the maximum value in the original data vector.

The normalized data were used to calculate the mutual information according to the channel, and Equation (4) represents the calculation method of the mutual information. Mutual information is a concept used in information theory to measure the correlation between two random variables. It is defined as the degree to which the uncertainty of one random variable is reduced, given that the value of the other is known. Mutual information can be used to measure the degree of correlation between two variables. When the mutual information is 0, it means that the two variables are independent. When the mutual information is greater than 0, it indicates a correlation between the two variables, and a higher amount of mutual information shows a higher correlation [27]. This study used mutual information to reduce the dimension of the channel and reduce the computational extent and the number of parameters.

(4) $$I\left(X;Y\right)=\sum_{x\in X}\sum_{y\in Y}p\left(x,y\right)log\frac{p(x,y)}{p\left(x\right)p(y)}$$

Here, *I*(*X*;*Y*) represents the joint probability distribution of *X* and *Y*, and *p*(*x*) and *p*(*y*) are the marginal probability distributions of *X* and *Y*, respectively.

### 3.6. Feature Extraction and Selection

After cutting the time window, the statistical and information theoretic features of the data in each time window were calculated, and these features expressed the HfNIRS signal. Table 1 describes the extracted features and feature descriptions.

The data of the 15 HfNIRS sensors were used, and each sensor had three indicators: Hb, HbO_2_, and Hbt. After feature screening, each data window sample had 32 × 9 features. Given the relationship between the number of pieces per participant and the number of instances, feature selection is essential to reduce the number of parameters trained in the classifier. As a feature selection method, this study used a recursive feature elimination algorithm, where, in each classification fold, only the training set was used for cross-validation to obtain features that optimized model performance.

### 3.7. Feature Classification

After determining the features, the KNN algorithm was used for classification in the machine learning algorithm. The basic principle of the method is as follows:

- Data preparation: First, it is necessary to prepare a labeled training dataset, including input samples and corresponding labels.
- Distance measure: The KNN algorithm uses distance measures to calculate sample similarity. Standard distance measures include Euclidean, Manhattan, and Minkowski [28].
- Selection of the K value: K in the KNN algorithm stands for the selection of the nearest K neighbors for decision making. A suitable value of K needs to be chosen and is usually determined by cross-validation.
- Neighbor selection: For the sample to be classified, the distance between it and each sample in the training set was calculated, and the nearest K samples were selected as neighbors.
- Majority voting: For the classification problem, the sample’s label is decided according to the neighbor’s label. Usually, the majority voting method is adopted; that is, the label with the most occurrences in the neighbors is selected as the sample label to be classified.
- Regression problem: For the regression problem, the average value of the neighbors can be used as the predicted value of the expected samples.

The advantage of the KNN algorithm is its simplicity: the KNN algorithm is intuitive and easy to understand. It does not need to assume the distribution of the data, nor does it need to carry out model training; it only needs to calculate the distance and select the nearest neighbor. It is applicable to a variety of data types. The KNN algorithm can be applied to all kinds of data, including discrete, continuous, categorical, and regressive data. Non-parametric learning: The KNN algorithm is a non-parametric learning method that does not make any assumptions about the distribution of the data. This enables KNN to adapt to irregular and complex data distribution. Strong interpretability: the prediction results of the KNN algorithm can be directly interpreted by the category or numerical value of the nearest neighbors. This makes the KNN algorithm more acceptable and understandable in some fields. Good effect on a small quantity of sample data: when the quantity of training data is small, the KNN algorithm can still show a good performance. This is useful for minor sample problems in some domains.

Accuracy rate, precision rate, recall rate, and F1 score are used to evaluate the performance of the model, and the corresponding expressions are as follows: (5)–(8), where TP, TN, FP, and FN represent true positive, true negative, false positive, and false negative, respectively. Taking the layout problem as an example, true positive (TP) means that the sample prediction is the layout problem, which corresponds to the layout problem. True negative (TN) indicates that the sample prediction is not a layout problem, which corresponds to a layout problem. False positive (FP) indicates that the sample is predicted as a non-layout problem, reaching the layout problem. A false negative (FN) means that the sample prediction is a layout problem corresponding to a non-layout problem [29].

(5) $$Accuracy=\frac{TP+TN}{TP+TN+FP+FN}$$

(6) $$Precision=\frac{TP}{TP+FP}$$

(7) $$Recall=\frac{TP}{TP+FN}$$

(8) $$F1-Score=\frac{2(Precision\cdot Recall)}{Precision+Recall}$$

## 4. Results

**Figure 7:**
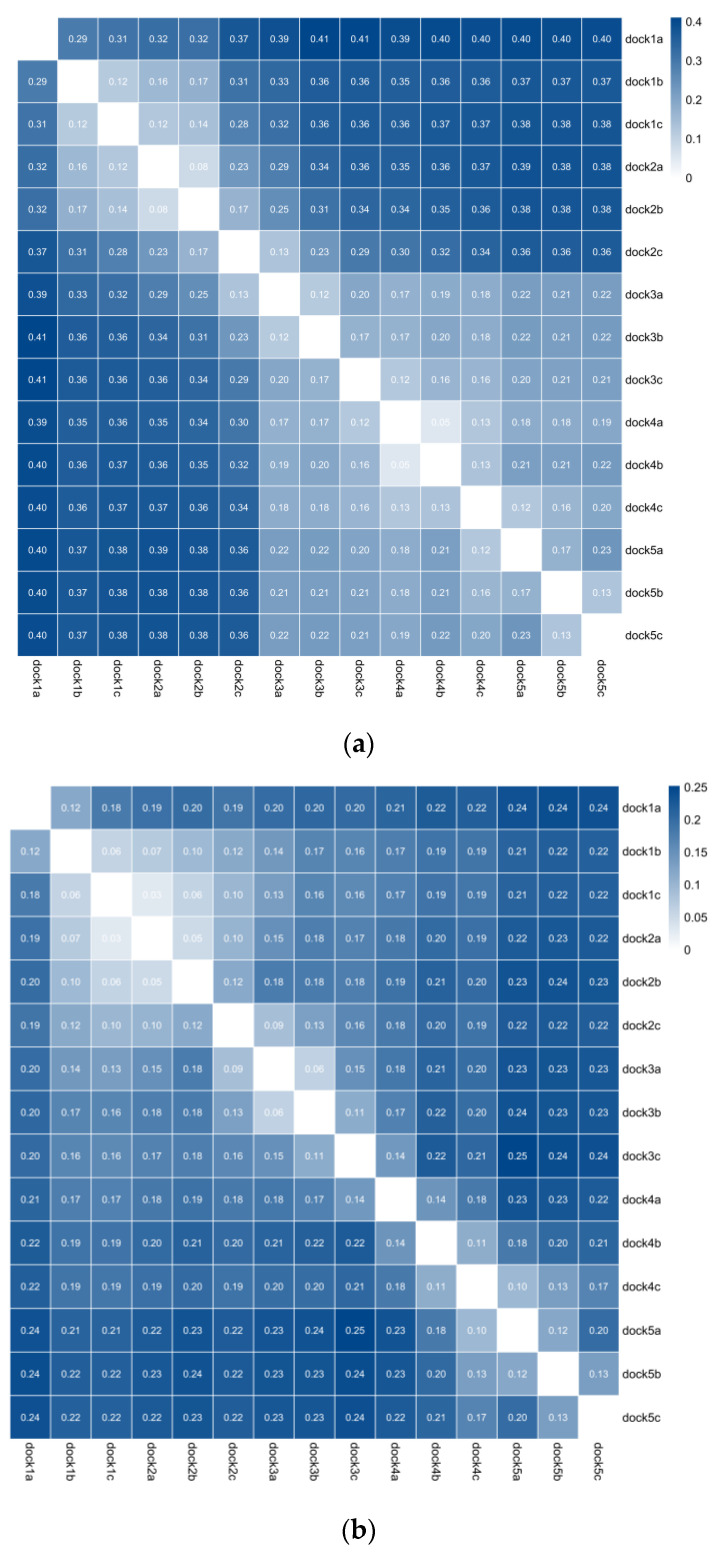

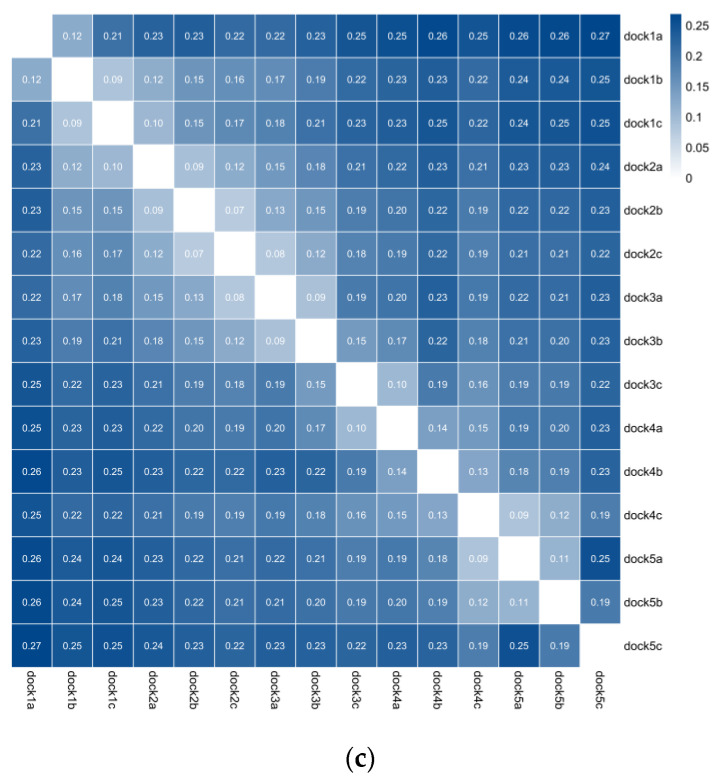
(**a**) represents the layout problem Hb channel mutual information graph; (**b**) represents the layout problem HbO_2_ channel mutual information graph; (**c**) represents the layout problem Hbt channel mutual information graph. dock1a represents the first channel of an optical sensor; the number represents the result of the mutual information; the darker the color, the greater the mutual information.

This study used five channels and fifteen groups of data from three optical sensors for analysis. Due to the high data dimension, the research and practical application process was more complicated. After preprocessing the data through normalization, the mutual information was calculated to screen the channels. Figure 7 takes the layout problem as an example to draw the channel mutual information diagram of Hb, HbO_2_, and Hbt. Through the mutual information diagram, there is a specific correlation between the data of the different channels and the different optical sensors. In the analysis process, the relevant media can retain a set of data for analysis; the channel results of the other problems are shown in Table 2. If the related studies consider analyzing the layout problem from the Hb perspective, they can focus on analyzing the data on the seven channels: 1b, 2b, 3a, 4a, 4c, 5a, and 5c. If the analysis ability is limited, we can focus on the data of 1b and 2b channels. The above seven channels or two channels have specific reference values for studying layout problems.

Each question contained four steps: question judgment, picture visualization, question answer, and question fixation point. The feature calculation and KNN classification were performed on the four links of the four questions, and the classifications accuracy, precision, recall, F1 score, and time were calculated, as shown in Table 3.

**Figure 8:**
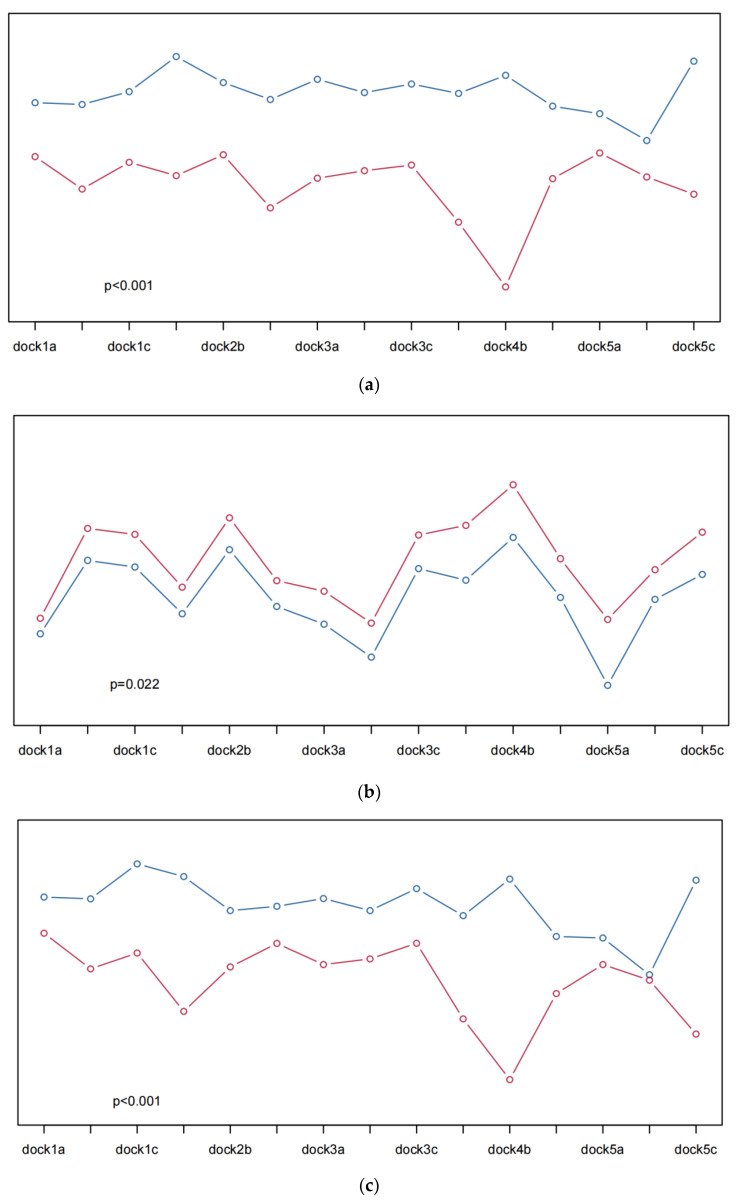
(**a**) shows the Hb fluctuations of volunteers when they answered the layout questions; (**b**) shows the HbO_2_ fluctuations of volunteers when they answered the layout questions; (**c**) shows the Hbt fluctuations of volunteers when they answered the layout questions. The ordinate of the image ranges from 0–1, indicating the value normalized by the relative concentration. Higher curves represent greater relative concentrations. Blue indicates Hb, HbO_2_, and Hbt fluctuations when the volunteer questionnaire scores are high, and red indicates low Hb, HbO_2_, and Hbt fluctuations when the volunteer questionnaire scores are low.

To better quantify the visual perception, the questionnaire scores were divided into two groups according to the average value, and the average values of Hb, HbO_2_, and Hbt in the different channels were plotted. The differences in the Hb, HbO_2_, and Hbt values between the high-score group and the low-score group were compared. Taking the layout problem as an example (Figure 8), all the *p* values were less than 0.05, indicating that there were significant differences in the Hb, HbO_2,_ and Hbt fluctuations between the high and low scores.

## 5. Discussion

To accomplish the quantification of visual perception in this project, this study took the following steps. First, this study collected Hb, HbO_2_, and Hbt data from many volunteers in the field of automobile design using HfNIRS technology; the data were obtained in different tasks and sections. Then, this study calculated 15 sets of data based on five channels and three optical sensors and used mutual information to reduce the dimension of these data. Next, this study extracted statistical and information theoretic features from these data, respectively. This study adopted the KNN classification algorithm to classify different tasks and links accurately. Finally, this study comprehensively analyzed the questionnaire results with the collected fluctuation data of Hb, HbO_2_, and Hbt and judged whether there were differences between Hb, HbO_2_, and Hbt under different scores. With these steps, this study successfully quantified visual perception.

In the actual data processing process, normalization is a crucial step that can enhance the model’s performance and stability by scaling the eigenvalues of different scales to the same scale. Specifically, normalization can avoid the dominant role of some features in model training so that the model treats each component more fairly, and it can also make the comparison between different features more intuitive. After normalization, the convergence speed of the model will also be significantly accelerated, and the interpretability of the model can be improved so that this study can understand the importance of different features more intuitively. In addition, normalization can also reduce the influence of outliers on the model so that the model is not disturbed by the outliers. Finally, the normalization process can also be adapted to the requirements of different algorithms so that this study can more easily apply various algorithms for model training and optimization. Normalization can help us to process the data better, improve the performance and stability of the model, reduce the influence of outliers, and adapt to the requirements of different algorithms. Therefore, when performing data processing, this study should carefully perform normalization to improve data processing efficiency and model training effect.

Mutual information is calculated by measuring the degree of interdependence between two variables to capture their relevance. This calculation method is unbiased; that is, it is not affected by the shape of the distribution of the variables, the noise, or the outliers; thus, it effectively avoids the limitations of traditional methods when dealing with nonlinear relationships. Therefore, the calculation of mutual information also has a wide range of applications in unsupervised learning and can help us better understand the interaction and importance of different variables in the data. Normalization of the data is usually required before mutual information calculation. After normalization, the analysis of mutual information can ensure the unity of the feature scales and avoid the influence of scale differences on the calculation results of the mutual information. This can also prevent the occurrence of bias, thereby improving the stability of the results. The interpretability of mutual information can help us to better understand the interaction and importance of different variables in the data. By calculating mutual information, this study can learn which variables are associated with each other, to what extent these are associated, and the impact of this association on the whole dataset. This information can help us to better understand the structure of the data and to provide strong support for subsequent analysis and prediction. The calculation of mutual information is a practical analysis method that can capture the association between variables, and it has the advantages of unbiasedness, interpretability, and stability. When dealing with complex data, the calculation of mutual information can help us to better understand the structure of the data and the relationship between different variables and to provide strong support for subsequent analysis and prediction.

Neural networks are an effective way to deal with high-dimensional data. However, in the actual analysis process, it is difficult to directly obtain solutions to practical problems due to the poor interpretation of neural networks. Therefore, channel dimension reduction combined with machine learning algorithms can better solve practical problems. Channel dimension reduction is an effective method that can reduce the computational load and improve the computational efficiency while reducing the risk of overfitting and enhancing the model’s generalization ability. In addition, channel dimension reduction can also enhance the interpretability of the model, reduce redundant information and noise, and improve the model’s effect. By reducing the number of channels, the model can be more focused on learning discriminative and valuable features, thereby enhancing the representation power and performance of the model. In addition, when combined with the results in Table 2, it is found that the dimension reduction of mutual information can change the number of channels after dimension reduction by limiting the threshold. This dimension reduction method plays a vital role in the subsequent classification algorithm because the choice of entry affects the effect of the classification algorithm. Therefore, in practice, choosing the appropriate threshold according to the specific problem is necessary to achieve the best classification effect. When combined with the analysis process, it is found that the channel dimensionality reduction combined with the machine learning algorithm is one of the effective methods for solving high-dimensional data processing. Channel dimension reduction can significantly improve computational efficiency and reduce the storage cost and memory footprint when performing inference or training tasks on resource-constrained devices. At the same time, attention should be paid to selecting the appropriate threshold for dimensionality reduction to achieve the best classification effect.

The assessment of visual perception is often influenced by subjective factors, making the differences between different individuals significant; thus, it is challenging to make an accurate analysis from an individual perspective. Therefore, to achieve accurate quantification of visual perception, a model with high applicability and accuracy must be constructed to eliminate the subjective influence. It can be seen from the classification results in Table 3 that the classification accuracy of different problems and different links reaches 96.3%, which indicates that the accuracy of the model is high. In addition, the classifier results showed apparent differences in the HfNIRS feedback in different sessions of different questions, suggesting that HfNIRS can be used as an effective means of quantifying visual perception. At the same time, by calculating the feedback time of additional questions and different links, combined with the *p* value results of hypothesis testing, this study found that all *p* values were less than 0.001. This means that in terms of the quantification of visual perception, it is also feasible from the perspective of feedback time. By constructing a model suitable for quantifying visual perception with high accuracy, this study can effectively eliminate the influence of subjectivity to achieve accurate visual perception quantification. This conclusion was validated in terms of the classification results and the HfNIRS feedback and further supported by the *p* value results. Therefore, using HfNIRS to quantify visual perception is feasible.

According to the visualization results in Figure 8, for the layout problem, the Hb, HbO_2_, and Hbt fluctuations with high scores in any channel are higher than those with low scores, and the *p* value is less than 0.05; that is, there is a significant difference. Regarding the judgment of the layout problem, the questionnaire data proved that HfNIRS can quantify the layout problem. The larger the value corresponding to HfNIRS, the higher the satisfaction with the layout problem. For the color problems, the 3b and 4c channels of Hb and HbO_2_ have high scores, but the fluctuation value of HfNIRS is low. The 3b, 4c, and 5a channels of Hbt have high scores, but the fluctuation value of HfNIRS is low. When combined with the picture color analysis, the proportion of blue in the picture is large. Blue is considered a calm, stable, and relaxing color, giving people a sense of security and trust, resulting in lower Hb, HbO_2_, and Hbt fluctuations. However, the *p* values were less than 0.05, indicating significant differences in HfNIRS under different scores. For the design and information problems, the Hb, HbO_2_, and Hbt fluctuations with high scores in any channel were higher than those with low scores, and the *p* value was less than 0.001; that is, there was a significant difference. Regarding the judgment of the design and information problems, the questionnaire data proved that HfNIRS could quantify the design problems and information problems. Moreover, the larger values corresponding to HfNIRS represented higher satisfaction with the design and information issues.

## 6. Conclusions

This work aims to quantify visual perception by combining the perspectives of statistics, information theory, and machine learning to reduce the dimensionality of HfNIRS channels. Visual perception is a complex cognitive process that includes many factors; so, comprehensive quantification is challenging. Nonetheless, the present study showed some results in quantifying visual perception using HfNIRS.

In this study, the fluctuation characteristics of different channels were statistically analyzed, the correlation analysis of information theory was combined, and the feature extraction and classification algorithms of machine learning were used to reduce the dimension of the HfNIRS channels. Specifically, the fluctuation characteristics of each track were first described and analyzed using statistical methods. Then, the information theory method explored the correlation between these channel fluctuations. Finally, a machine learning algorithm was used to classify and identify the channel fluctuations to quantify visual perception.

However, due to the limitations of this study, such as the limitation of the experimental pictures and the population, the quantification of visual perception could only be partially achieved. In the future, more photos with different colors can be considered for more accurate quantification of visual perception, and the relationship between channel fluctuations can be further explored.

This study uses HfNIRS to quantify visual perception, hoping to provide reliable evaluation data for subsequent automotive-related graphic design or texture design. The subsequent development of the evaluation system in the automotive industry will further verify the effectiveness of HfNIRS.

In conclusion, this work has achieved some results in quantifying visual perception using HfNIRS, but it still needs further improvement and perfection. In the future, this study can expand the experimental pictures and populations to distinguish the optical perception channels and characteristics of different working people. Attempts were made to extract more accurate depth features from different perspectives. Multi-modal fusion quantification was achieved by integrating various indicators. Other classification methods combined with the proposed idea were explored to give more accurate results.

## Figures and Tables

**Table 1:** Features and feature descriptions.

Domain	Feature	Description
	Time	The overall average time of the data.
Statistical	Mean	The overall average of the data.
	Median	The overall level of data was moderate.
	Variance	The degree to which the overall relative average of the data deviates.
	Range	The overall number of variations in the data.
	Kurtosis	The steepness of the overall probability distribution of the data.
	Deviation	The degree of symmetry of the overall probability distribution of the data.
Entropy	Information entropy	The total amount of information contained in the data.
	Approximate entropy	The overall irregularity and complexity of the data.

**Table 2:** Channel dimension reduction results; 1a represents the first channel of an optical sensor.

Problem	Hb	HbO_2_	Hbt	Common Channel
Layout problem	1b, 2b, 3a, 4a, 4c, 5a, 5c	1b, 1c, 2b, 3b, 4b, 5a, 5b	1b, 2b, 3a, 4a, 4b, 4c, 5b	1b, 2b
Design problem	1b, 1c, 2b, 3a, 4a, 4c, 5a	1b, 1c, 2c, 3a, 4b, 5a, 5c	1b, 1c, 2b, 3b, 4a, 4c, 5b	1b, 1c
Color problem	1b, 2a, 2c, 3b, 4c, 5a, 5b	1a, 1c, 2c, 4a, 4b, 5a, 5c	1b, 2a, 2c, 3b, 4b, 4c, 5a	2c, 5a
Information problem	1b, 2a, 2c, 3a, 3c, 5a, 5c	1b, 1c, 2c, 3b, 3c, 4b, 5b	1b, 1c, 2b, 3a, 4a, 4c, 5c	1b

**Table 3:** Classification results of each link in the experimental process. All results are presented as percentages.

Participant	Accuracy	Precision	Recall	F1 Score	Time
Layout judgment	99.3	99.0	98.1	98.5	201.6 s
Layout diagram	96.1	97.2	92.3	94.7	241.2 s
Layout problem	96.5	98.6	95.7	97.1	163.1 s
Layout fixation point	97.2	98.6	95.8	97.2	314.4 s
Color judgment	98.0	98.6	96.0	97.3	145.6 s
Color diagram	95.3	97.2	92.0	94.5	242.4 s
Color problem	95.7	98.6	95.4	97.0	161.8 s
Color fixation point	97.2	98.6	95.8	97.2	289.8 s
Design Judgment	99.9	99.9	99.9	99.9	186.1 s
Design diagram	98.0	99.9	99.9	99.9	252.2 s
Design problem	91.4	93.1	82.8	87.6	170.0 s
Design fixation point	95.7	98.6	95.4	97.0	290.4 s
Information judgment	98.0	98.6	96.0	97.3	223.5 s
Information diagram	95.3	95.8	92.0	93.9	245.4 s
Information problem	96.5	91.7	95.6	93.6	159.3 s
Information fixation point	99.3	95.8	98.1	96.9	316.7 s
Average	96.3 ± 1.99	97.5 ± 2.25	95.1 ± 3.92	96.2 ± 2.86	225.2 ± 56.0

## Data Availability

At this moment, the data are only available from the corresponding author upon request as they are being prepared for publication.
